# *BreastMark*: An Integrated Approach to Mining Publicly Available Transcriptomic Datasets Relating to Breast Cancer Outcome

**DOI:** 10.1186/bcr3444

**Published:** 2013-07-02

**Authors:** Stephen F Madden, Colin Clarke, Patricia Gaule, Sinead T Aherne, Norma O'Donovan, Martin Clynes, John Crown, William M Gallagher

**Affiliations:** 1 Molecular Therapeutics for Cancer Ireland, National Institute for Cellular Biotechnology, Dublin City University, Glasnevin, Dublin 9, Ireland; 2 UCD School of Biomolecular and Biomedical Science, UCD Conway Institute, University College Dublin, Dublin 4, Ireland

## Abstract

**Introduction:**

Breast cancer is a complex heterogeneous disease for which a substantial resource of transcriptomic data is available. Gene expression data have facilitated the division of breast cancer into, at least, five molecular subtypes, namely luminal A, luminal B, HER2, normal-like and basal. Once identified, breast cancer subtypes can inform clinical decisions surrounding patient treatment and prognosis. Indeed, it is important to identify patients at risk of developing aggressive disease so as to tailor the level of clinical intervention.

**Methods:**

We have developed a user-friendly, web-based system to allow the evaluation of genes/microRNAs (miRNAs) that are significantly associated with survival in breast cancer and its molecular subtypes. The algorithm combines gene expression data from multiple microarray experiments which frequently also contain miRNA expression information, and detailed clinical data to correlate outcome with gene/miRNA expression levels. This algorithm integrates gene expression and survival data from 26 datasets on 12 different microarray platforms corresponding to approximately 17,000 genes in up to 4,738 samples. In addition, the prognostic potential of 341 miRNAs can be analysed.

**Results:**

We demonstrated the robustness of our approach in comparison to two commercially available prognostic tests, onco*type * DX and MammaPrint. Our algorithm complements these prognostic tests and is consistent with their findings. In addition, *BreastMark * can act as a powerful reductionist approach to these more complex gene signatures, eliminating superfluous genes, potentially reducing the cost and complexity of these multi-index assays. Known miRNA prognostic markers, mir-205 and mir-93, were used to confirm the prognostic value of this tool in a miRNA setting. We also applied the algorithm to examine expression of 58 receptor tyrosine kinases in the basal-like subtype, identifying six receptor tyrosine kinases associated with poor disease-free survival and/or overall survival (EPHA5, FGFR1, FGFR3, VEGFR1, PDGFRβ, and TIE1). A web application for using this algorithm is currently available.

**Conclusions:**

*BreastMark * is a powerful tool for examining putative gene/miRNA prognostic markers in breast cancer. The value of this tool will be in the preliminary assessment of putative biomarkers in breast cancer. It will be of particular use to research groups with limited bioinformatics facilities.

## Introduction

Breast cancer is a complex heterogeneous disease which has traditionally been subclassified depending, amongst other factors, on the expression of different receptor proteins, such as estrogen receptor (ER), progesterone receptor (PR), and human epidermal growth factor receptor 2 (HER2) [1]. These 'biomarkers' allow us to tailor the level of clinical intervention. While ER-positive **the second positive should be deleted **tumours receive hormone therapies [2] and HER2-positive cancers receive targeted therapies such as trastuzumab and lapatinib [3], 'triple negative' cancers lacking these markers currently have no targeted therapies and cause a disproportionate number of breast cancer deaths [4]. In addition to the traditional classifications using these biomarkers, in recent years, whole genome DNA microarrays have been utilised to further classify this disease, initially into five molecular subtypes based on gene expression profiles, namely luminal A and luminal B (ER-positive tumours), HER2 (HER2-positive tumours), basal and normal-like tumours [5,6] and subsequently into at least ten further molecular subtypes using both copy number and gene expression data [7].

It is crucially important to identify which breast cancer patients are at risk of developing a more aggressive phenotype so as to tailor the level of clinical intervention. Prognostic biomarkers, such as ER and HER2, can be used to assess the inherent likelihood of a patient exhibiting a particular outcome. However, within the subtypes defined by these classical markers, there is a wide spectrum of survival requiring the identification of additional novel prognostic markers. Also, the triple negative subtype has no such prognostic biomarkers currently in clinical use.

There is a great deal of transcriptomics data currently available to facilitate the identification of novel molecular biomarkers associated with breast cancer and its subtypes. Huge studies such as the 2,000 breast tumour profiles by Curtis *et al*. [7] greatly aid in our understanding of breast cancer and facilitate the identification of novel intrinsic subtypes. The diverse nature of these datasets and the variability of the different microarray platforms themselves can affect the statistical power of such studies. Moreover, it is necessary to test the prognostic ability of markers in diverse datasets to avoid dataset-specific affects.

It is clear that the selection of markers could benefit greatly from the integration of datasets from multiple studies to increase confidence in the selected markers. To this end, we have developed an easy-to-use interface for our algorithm which allows identification of subsets of genes that are associated with disease progression in breast cancer or its subtypes, that is, a set of putative prognostic markers. This algorithm integrates gene expression data from DNA microarray studies and corresponding clinical data (hormone status, survival time, tumour grade, patient age and so on). In particular, it allows investigation of prognostic markers in the context of disease-free survival (DFS), distant disease-free survival (DDFS) and overall survival (OS).

Over the last decade, our understanding of the function that small non-coding RNAs known as microRNAs (miRNAs) play in an array of fundamental biological processes in both plants and animals has increased dramatically [8]. These short endogenous non-coding RNAs act primarily by negatively regulating the expression of target mRNAs through translational inhibition and/or mRNA degradation [8]. The complexity of post-transcriptional control of gene expression by miRNAs remains a significant challenge. Indeed, miRNAs have the potential to alter entire pathways due to their ability to target multiple genes simultaneously [9]. The association of miRNAs with breast cancer has been well established [10,11]. In fact, miRNAs have been identified as prognostic markers in breast cancer [12] and associated with breast tumours defined by their HER2 or ER/PR status [13].

Approximately 50% of known human miRNAs are intronic (miRBase release 18, November 2011). Of these, 341 or roughly one third of human miRNA host genes are hybridized by probes on the U133plus2 Affymetrix gene chip. A number of studies have reported that many intronic miRNAs show significantly correlated expression profiles with their host genes [14,15]. Estimates of the number of miRNAs whose expression profiles are significantly correlated with their host gene are as high as 70% [16]. The expression of these miRNAs can, in some instances, be inferred from the expression of their host genes and can, therefore, be evaluated as putative prognostic markers in breast cancer and its subtypes using gene expression data.

We evaluated our approach using two commercially available gene expression-based prognostic tests in breast cancer, namely onco*type *DX and MammaPrint. We also applied the algorithm to examine the expression of 58 receptor tyrosine kinases (RTKs) in the basal-like subtype of breast cancer. Using the 21 genes from onco*type *DX and the 70-gene MammaPrint signature, we demonstrated the robustness of our approach and confirmed the prognostic value of these signatures. In the case of onco*type *DX, we showed that the predictive strength of this test is centred on the five proliferation genes within the 21 gene set. We also identified six RTKs associated with poor prognosis in the basal breast cancer subtype. The feasibility of using miRNA host gene expression as a surrogate for miRNA levels was tested using known miRNA prognostic markers, mir-93 and mir-205. Although these markers were only identified in small patient cohorts, *BreastMark *was able to confirm the robustness of these prognostic markers across a far larger and diverse patient dataset. A web application for using this algorithm is currently available [17].

## Methods

### Gene expression data

Gene expression data sets were downloaded from the Gene Expression Omnibus [18] or authors' websites in the form of raw data files, where possible. Only breast cancer datasets with survival information and at least 48 patients were included. Large datasets were chosen for this analysis so as to avoid the sampling effects associated with small datasets. A cut-off of 48 was chosen as all smaller breast cancer datasets either lacked detailed clinical data or had too few samples (approximately 30 samples or less). In total, 4,738 samples across 26 datasets incorporating 12 different microarray platforms were utilised to develop the *BreastMark *system (Table 1). Table 2 contains a breakdown of the clinical information available with each dataset. Where raw data were not available, the normalised data as published by the original authors were used. In the case of the raw data for the Affymetrix datasets (.cel files), gene expression values were called using the **GeneChip (GC) **robust multichip average method [19] and data were quantile normalised using the Bioconductor package, affy [20]. For the dual-channel platforms, data were loess normalised [21] using the Bioconductor package limma. Hybridisation probes were mapped to Entrez gene IDs to gene centre the data [22]. The Entrez gene IDs corresponding to the array probes were obtained using Biomart [23,24] and the Bioconductor annotation libraries. Probes that hit multiple genes were filtered out. If there were multiple probes for the same gene, the probe values were averaged for that gene. This resulted in expression data for a total of 20,017 Entrez gene IDs across 4,738 samples.

**Table 1:** Datasets used in this analysis

GEO Accession Number	Reference	Data Format	Sample Number	Platform Type (probe number)
GSE7849	Anders *et al*., 2008 [51]	Processed only	78	Affymetrix Human Genome U95 Version 2 Array (12,625 probes)
GSE3143	Bild *et al*., 2006 [52]	Raw .CEL files	158	Affymetrix Human Genome U95 Version 2 Array (12,625 probes)
GSE12276	Bos *et al*., 2009 [53]	Raw .CEL files	204	Affymetrix U133 Plus 2.0 (54,675 probes)
GSE22219	Buffa *et al*., 2011 [44]	Raw Data files	216	Illumina humanRef-8 v1.0 expression beadchip
GSE10510	Calabro *et al*., 2009 [54]	Raw .gpr files	152	DKFZ Division of Molecular Genome Analysis Human Operon 4.0 oligo Array 35 k (36,486 probes)
NA	Chang *et al*., 2005 [31]	Processed only	295	Agilent 21 K oligo array (22,575 probes)
NA	Chin *et al*., 2006 [55]	Processed only	118	Affymetrix U133AAofAv2 (22,944 probes)
GSE9893	Chanrion *et al*., 2008 [56]	Raw data available	155	MLRG Human 21 K V12.0 (22,656 probes)
GSE7390	Desmedt *et al*., 2007 [57]	Raw .CEL files	198	Affymetrix U133A (22,283 probes)
GSE16391	Desmedt et *al*., 2009 [58]	Raw .CEL files	48	Affymetrix U133 Plus 2.0 (54,675 probes)
GSE25055	Hatzis *et al*., 2011 [59]	Raw .CEL files	508	Affymetrix U133A (22,283 probes)
GSE24450	Heikkinen *et al*., 2011 [60]	Raw Data files	183	Illumina HumanHT-12 V3.0 expression beadchip
GSE1992	Hu *et al*., 2006 [27]	Processed only	99	Agilent 21 K oligo array (22,575 probes)
GSE20685	Kao *et al*., 2011 [61]	Raw .CEL files	327	Affymetrix U133 Plus 2.0 (54,675 probes)
NA	Kok *et al*., 2009 [62]	Processed only	109	Agilent 44 K oligo array (54,675 probes)
GSE9195	Loi *et al*., 2008 [63]	Raw .CEL files	77	Affymetrix U133 Plus 2.0 (54,675 probes)
GSE6532	Loi *et al*., 2008 [63]	Raw .CEL files	265	Affymetrix U133A/B (22,283/22,645 probes) and U133 Plus 2.0
GSE1378, GSE 1379	Ma *et al*., 2004 [64]	Processed only	60	Custom 22 K oligo array (22,575 probes)
GSE3494	Miller *et al*., 2005 [65]	Raw .CEL files	251	Affymetrix U133A/B (22,283/22,645 probes)
GSE45255	Nagalla *et l*., 2013 [66]	Raw .CEL files	139	Affymetrix U133A (22,283 probes)
GSE1456	Pawitan *et l*., 2005 [67]	Raw .CEL files	159	Affymetrix U133A/B (22,283/22,645 probes)
GSE21653	Sabatier *et al*., 2010 [68]	Raw .CEL files	266	Affymetrix U133 Plus 2.0 (54,675 probes)
GSE11121	Schmidt *et al*., 2008 [69]	Raw .CEL files	200	Affymetrix U133A (22,283 probes)
GSE17907	Sircoulomb *et al*., 2010 [70]	Raw .CEL files	51	Affymetrix U133 Plus 2.0 (54,675 probes)
GSE2034	Wang *et al*., 2006 [71]	Raw .CEL files	286	Affymetrix U133A (22,283 probes)
GSE12093	Zhang *et al*., 2008 [72]	Raw .CEL files	136	Affymetrix U133A (22,283 probes)
	Total		4738	

**Table 2:** Clinical data summary

GEO ID	Median age	Median size (cm)	Lymph node status	**Chemo-therapy info**.	**Hormone treatment info**.	ER status	HER2 status	PR status	Tumour grade (1/2/3)	DFS (months)	DDFS (months)	OS (months)
GSE7849	55 ± 12	2.3 ± 1.1	A	A	A	A	NA	A	2/30/34	81 ± 40	NA	NA
GSE3143	NA	NA	NA	NA	NA	NA	NA	NA	NA	51 ± 31	NA	A
GSE12276	NA	NA	NA	NA	NA	NA	NA	NA	NA	26 ± 22	NA	NA
GSE22219	55 ± 11	2.6 ± 1.4	A	NA	NA	A	NA	NA	41/87/63	94 ± 38	NA	NA
GSE10510	59 ± 12	NA	A	NA	NA	A	NA	A	NA	57 ± 53	NA	87 ± 60
NKI295, (Chang *et al*., 2005)	44 ± 5	2.25 ± 0.9	A	A	NA	A	NA	NA	NA	84 ± 50	NA	94 ± 47
Chin *et al*., 2006	55 ± 15	2.7 ± 1.4	A	A	A	A	A	A	10/42/61	NA	69 ± 48	NA
GSE9893	67 ± 10	2.3 ± 0.9	A	NA	A	A	NA	NA	21/94/33	65 ± 32	66 ± 31	72 ± 29
GSE7390	46 ± 7	2.2 ± 0.8	NA	NA	NA	A	NA	NA	30/83/83	113 ± 68	114 ± 65	138 ± 61
GSE16391	62 ± 8	NA	A	A	A	A	A	A	NA	35 ± 15	NA	NA
GSE25055	49 ± 10	NA	A	A	A	A	A	A	32/180/259	NA	36 ± 20	NA
GSE24450	NA	NA	NA	NA	NA	NA	NA	NA	NA	NA	NA	72 ± 27
GSE1992	55 ± 15	NA	A	NA	NA	A	NA	NA	8/34/57	25 ± 23	NA	29 ± 25
GSE20685	NA	NA	NA	NA	NA	NA	NA	NA	NA	NA	88 ± 43	94 ± 38
Kok *et al*., 2009	NA	NA	NA	NA	NA	NA	NA	NA	NA	15 ± 17	NA	NA
GSE9195	64 ± 9	2.4 ± 0.96	A	NA	A	A	NA	A	14/20/24	95 ± 30	97 ± 28	NA
GSE6532	59 ± 13	2.2 ± 0.9	A	NA	A	A	NA	A	38/71/24	71 ± 42	71 ± 42	NA
GSE1378, GSE1379	67 ± 9	2.3 ± 1.1	A	NA	NA	A	A	A	3/39/18	87 ± 46	NA	NA
GSE3494	62 ± 13	2.3 ± 1.25	A	NA	NA	A	NA	A	67/128/54	NA	NA	98 ± 46
GSE45255	55 ± 12	2.9 ± 1.3	A	A	A	A	A	A	17/52/67	48 ± 22	51 ± 25	54 ± 21
GSE1456	NA	NA	NA	NA	NA	NA	NA	NA	28/58/61	72 ± 29	NA	77 ± 23
GSE21653	54 ± 14	NA	A	NA	NA	A	A	A	45/89/125	60 ± 41	NA	NA
GSE17907	50 ± 14	NA	A	NA	NA	A	A	A	3/10/34	39 ± 29	NA	NA
GSE11121	NA	2 ± 0.99	A	NA	NA	NA	NA	NA	29/136/35	NA	94 ± 51	NA
GSE2034	NA	NA	A	NA	NA	A	NA	NA	NA	78 ± 42	NA	NA
GSE12093	NA	NA	A	A	A	A	NA	NA	NA	92 ± 38	NA	NA

A, available; ER, estrogen receptor; HER2, human epidermal growth factor receptor 2' PR, progesterone receptor; DFS, disease free survival; DDFS, distant disease free survival; NA, not available; OS, overall survival; tumour grade (1/2/3), 1 refers to number of grade 1 tumours, 2 refers to the number of grade 2 tumours and 3 refers to the number of grade 3 tumours.

### microRNA expression data

miRNAs are frequently located within the introns of protein coding genes and in exons of non-coding transcripts. miRNA expression can be detected using conventional microarrays through host gene expression for intragenic miRNAs or by direct probe matching for intergenic miRNAs. A total of 1,987 samples were processed on U133A Affymetrix arrays, while 973 were processed on U133plus2 Affymetrix arrays (2,960 in total). U133A and U133plus2 microarrays have 22,277 probe sets in common. Using this information, it is possible to infer the expression of 341 miRNAs across 2,960 samples [25] (based on miRBase version 13.0, Ensembl version 54_36p). As with the gene centred data, this information was also combined with the available clinical data for survival analysis.

### Breast cancer subtypes

The R package genefu [26] was used to classify the 4,739 breast cancer samples into the luminal A, luminal B, HER2, normal-like and basal molecular subtypes using the ssp2003 [5], ssp2006 [27], and pam50 [28], classifiers.

### Survival analysis

We have combined detailed clinical data from each of the studies used here, including one or more of DFS, DDFS and OS. The software allows for each of these three survival end points to be analysed separately. Median expression was used to dichotomise the data, allowing stratification into high and low groups within each of the 26 individual datasets. Once a sample was assigned to a particular group, the 26 datasets were combined and a global pooled survival analysis was performed in real-time. It is important to treat each dataset separately when determining which group a sample belongs to, as the expression of these genes will vary greatly across the different experiments/platforms. In essence, each dataset is split into high and low in singularity to negate study-specific effects. Survival curves are based on Kaplan-Meier estimates and the log-rank *P*-value is shown for difference in survival. Cox regression analysis was used to calculate hazard ratios. The R package 'survival' was used to calculate and plot the Kaplan-Meier survival curve. All calculations were carried out in the R statistical environment [29].

### Software parameters

The software incorporates all the clinical data made available by the original authors. This allows the data to be analysed based on one or more common clinical parameters including patient age, tumour size, lymph node status, tamoxifen treatment, chemotherapy treatment, ER status, HER2 status, PR status and tumour grade. The software also allows the upper or lower quartiles of the expression of the gene of interest to be used to determine high and low groups within each of the 26 individual datasets.

### Web server

The interface is available on a publicly accessible web server [17] and is updated quarterly. The software uses CGI to link the web server with the R/perl based algorithm. All calculations are carried out in real-time.

### Validation of BreastMark using the Onco*type *DX gene signature

The 21 gene signature used by onco*type *DX in predicting patient prognosis was downloaded from the original paper [30]. This panel of prospectively selected genes comprises 16 prognostic genes normalised relative to the expression of five reference genes. The 16 prognostic genes are broken down into five categories: proliferation, invasion, HER2, estrogen and 'other'. The likelihood of breast cancer relapse in patients was based on a recurrence score (RS) algorithm constructed and tested on a cohort of 668 patient samples. The higher the RS, the poorer the patient outcome observed. This algorithm weights each of the five categories based on the influence they have on disease recurrence. For example, the proliferation group is weighted most highly and, therefore, the expression of these genes influences the RS the most. Each of the 16 oncogenes were queried in our dataset to test the effect each gene has on survival using the three above-mentioned survival end-points for prognosis, namely DFS, DDFS and OS. It is expected that the genes with the greatest influence on the RS would have the highest hazard ratios and the lowest *P*-values. Sample numbers will vary depending on the number of platforms with expression information available for a particular gene.

### Validation of BreastMark using the MammaPrint gene signature

The 70-gene prognostic signature was downloaded from the original paper along with their correlation with prognosis [31]. It was possible to obtain unique Entrez gene IDs for 61 of these genes (there is more than one copy of PEC1, IGFBP5 and DIAPH3 (three) in the 70 gene list and five others have no Entrez gene ID). As with the onco*type *DX signature, each gene was analysed separately within our datasets using the three survival endpoints, DFS, DDFS and OS. Although looking at these genes individually does not represent the full power of this prognostic signature, this dataset should still be enriched for prognostic markers. Additionally, the positive and negative correlation coefficients published by the original authors should be consistent with our observed hazard ratios of less than or greater than 1, respectively. Sample numbers will vary depending on the number of platforms with expression information for a particular Entrez Gene ID.

### Receptor tyrosine kinases

We compiled a list of 58 RTKs from the literature. Using our algorithm, we identified which of the RTKs were associated with survival within the basal molecular subtype using the ssp2003, ssp2006 and PAM50 molecular classifiers (see above). DFS, DDFS and OS were used as the survival endpoints. A *P*-value of < 0.05 in a minimum of two out of three classifiers was considered significant. The data were dichotomised using three cut-offs, median expression, greater than the 75th percentile referred to as the 'high' cut-off and less than the 25th percentile referred to as the 'low' cut-off.

## Results

In order to test our gene-centred survival meta-analysis, we looked at the genes used to predict breast cancer prognosis by two commercially available tests, onco*type *DX [32] and MammaPrint [33]. Although the genes in these tests are not used in isolation to predict disease outcome, it is reasonable to assume that the genes chosen within these tests would include a number of prognostic markers whose expression in our meta-analysis would correlate with good and poor outcome. As there is currently no large-scale robust signature for miRNAs in breast cancer, we tested our approach on known individual miRNAs which have previously been shown to be prognostic markers. All calculations were carried out using the BreastMark web application [17].

### The robustness of BreastMark is tested using the 21 genes from Onco*type *DX

Onco*type *DX is a 21-gene signature (16 oncogenes and five controls) selected using prior knowledge from the literature, which in combination with the developer's algorithm, predicts patient outcome in lymph node-negative (LNN), ER-positive breast cancer [32]. It uses a RS calibrated against approximately 670 patients with known clinical outcome to predict patient survival. Patients with a low score do well, and those with a high score do poorly. The 16 genes are classified into five groups: proliferation, invasion, HER2, ER and other. The algorithm takes gene expression data from 16 oncogenes, normalises the expression against the five controls and weights the 16 oncogenes depending on the effect they have on the RS. The genes are weighted as follows 1.04 × proliferation group score + 0.47 × HER2 group score - 0.34 × ER group score + 0.1 × invasion group score + 0.05 × CD68 score - 0.08 × GSTM1 score - 0.07 × BAG1 score. Genes from the proliferation group, such as Ki67 and Survivin, have the highest weighting and, therefore, the greatest effect on the RS.

Each of the 16 oncogenes were analysed separately within *BreastMark *using median expression as a cut-off, selecting LNN, ER-positive patients only and using DFS survival as the survival end point to ensure comparability. This information is summarised in Table 3, along with the effect they have on the RS. The 16 genes were also analysed using DDFS and OS as the survival end points [see Additional file 1 Tables S1 and S2] and are consistent with our observations for DFS survival. A hazard ratio (HR) of greater than 1 indicates a negative effect on survival and a HR of less than one has a positive effect. The higher the HR the greater the effect the gene has on survival. As can be seen from Table 3, our results are largely consistent with the weightings calibrated for onco*type *DX. The proliferation markers which have the highest weightings, and therefore the largest effect on the RS, have the highest HRs and are highly statistically significant. In contrast, those genes which have only a marginal effect on the RS (CD68, GSTM1 and BAG1) are not significant and have HRs close to one.

**Table 3:** *BreastMark *results for the Onco*type *DX 21-gene signature for LNN, ER-positive patients using DFS as the survival end point

Onco*type *DX category	Gene symbol	*BreastMark* hazard ratio	*BreastMark *HR *P*-value	Sample number	RS weighting
Proliferation	KI67	1.68	4.40e-05	902	+1.04
	STK15	2.32	3.93e-11	902	
	Survivin	1.96	8.56e-08	902	
	CCNB1	1.89	3.63e-06	793	
	MYBL2	1.76	8.01e-06	902	
Invasion	MMP11	1.55	1.00e-03	875	+0.1
	CTSL2	1.42	7.12e-03	875	
HER2	GRB7	1.26	0.07	902	+0.47
	HER2	1.03	0.83	875	
ER	ER	1.32	0.05	875	-0.34
	PGR	0.80	0.08	902	
	BCL2	0.75	0.03	875	
	SCUBE2	0.71	0.03	628	
Other	GSTM1	0.92	0.56	651	-0.08
	CD68	0.96	0.74	902	+0.05
	BAG1	1.01	0.91	902	-0.07

**Figure 1:**
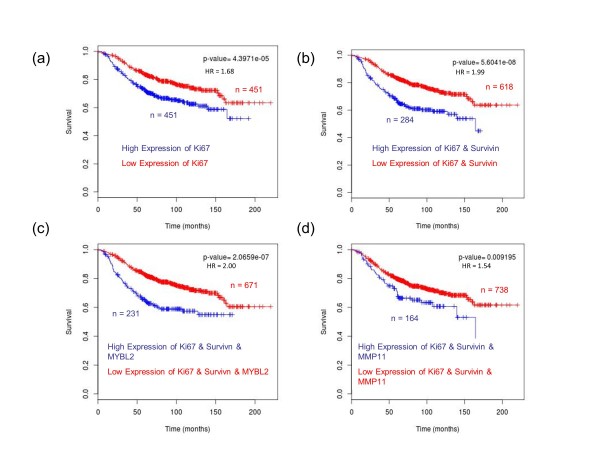
**Prognostic role of the Ki67, Survivn MYBL2 and MMP11 in breast cancer**. These figures were generated using *BreastMark *at http://glados.ucd.ie/BreastMark/index.html. (**a**) Kaplan-Meier estimates of survival, demonstrating high expression of Ki67 is associated with poor prognosis in breast cancer (*n *= 902, HR = 1.68, *P *= 4.44e-05). (**b**) Kaplan-Meier estimate of survival, demonstrating that high expression of Ki67 and Survivin in combination have a greater effect on survival (*n *= 902, HR = 1.99, *P *= 5.60e-08). (**c**) Kaplan-Meier estimate of survival, demonstrating that high expression of Ki67, Survivin and MYBL2 in combination have an even greater effect on survival (*n *= 902, HR = 2.00, *P *= 2.01e-07). (**d**) Kaplan-Meier estimate of survival, demonstrating how the invasion marker MMP11 does not improve the prognostic ability of Ki67 and Survivin (*n *= 902, HR = 1.54, *P *= 9.20e-03). HR, hazard ratio.

HR, hazard ratio; RS, Relapse score,

Combining the markers (grouping samples where both markers have greater than median expression) identifies patients who will do particularly poorly. The Kaplan-Meier plot for Ki67 in shown in Figure 1(a) (*n *= 902, HR = 1.68, *P *= 4.44e-05). The Kaplan-Meier plot for Ki67 and Survivin combined, that is, comparing the survival of patients with greater than median expression of both Ki67 and Survivin against the rest is shown in Figure 1(b). These patients have a worse prognosis than Ki67 alone, that is, they have a higher HR (a HR of 1.99 versus a HR of 1.68). The same occurs when you also combine MYBL2 with Ki67 and Survivin (Figure 1(c)). These patients have an even worse prognosis with an even greater HR (*n *= 902, HR = 2.00, *P *= 2.01e-07). However, the same is not true when you combine other markers with the proliferation markers. Figure 1(d) shows Ki67, Survivin and PGR combined (*n *= 902, HR = 1.537, *P *= 9.2e-03). The HR is lower and the difference in survival is less significant. In fact, when you combine most of the other oncogenes from the signature, no improvement in prognostic power or decrease in the significance of the HR is observed (data not shown). This suggests that not only are all of the genes from this prognostic signature not necessary, but that potentially our algorithm provides a useful reductionist approach to these more complex prognostic signatures, allowing us to eliminate superfluous markers and highlight those genes that are of the greatest relevance.

### BreastMark is consistent with the MammaPrint gene signature

Similar to the onco*type *DX assay, MammaPrint [33] is a commercially available test for breast cancer recurrence. In contrast, it was developed via a hypothesis-free method from a gene expression profiling study rather than from a prospectively chosen list of known oncogenes. The study used 78 LNN patients specifically to identify a prognostic signature in their gene expression profiles using a supervised classification method. Each of the approximately 25,000 probesets present on those microarrays were correlated with disease outcome and only those genes that were significantly associated with disease outcome were retained to create an optimised list of prognostic markers. Each of the 70 genes had a positive or negative correlation coefficient depending on their association with good or poor prognosis, respectively.

Again, as with onco*type *DX, even though the genes from the 70-gene signature are not predicted to act independently, the 70 genes when analysed independently, should correlate with good and poor prognosis based on the correlation coefficients identified in the original MammaPrint study. Genes with positive and negative correlation coefficients should have HRs less than and greater than one, respectively. As we expect, this is what we see with these genes in LNN samples, using a median cut-off and DFS survival as the survival endpoint (DDFS and OS show similar results in Additional file 1 Tables S3 and S4, respectively). Of the 61 genes from the MammaPrint signature for which we had Entrez gene IDs, 53 had HRs consistent with the correlation coefficients from the original study (Table 4). Of the other eight genes, four had HRs close to 1, and were not statistically significant, and the other four were not present in the dataset or present in too few samples. Although not all of the 53 consistent genes were statistically significant, 33 are significantly associated with survival when analysed independently with *BreastMark*.

**Table 4:** *BreastMark *results for the MammaPrint gene signature for LNN patients using DFS as the survival end point

Entrez Gene ID	Gene symbol	Hazard ratio	*P*-value	Sample number	MammaPrint correlation with prognosis
Good Prognosis					

8659	ALDH4	0.92	0.42	1105	0.421
8817	FGF18	0.86	0.16	1183	0.411
27113	BBC3	0.76	0.03	1004	0.407
57593	KIAA1442	NA	NA	NA	0.402
57758	CEGP1	0.69	5.37e-03	819	0.400
146923	RUNDC1	0.53	2.23e-03	387	0.390
8840	WISP1	0.85	0.13	1183	0.384
2947	GSTM3	0.79	0.02	1183	0.380
151126	ZNF533	0.84	0.39	382	0.375
146760	RTN4RL1	0.84	0.45	281	0.374
10455	PECI	0.81	0.05	1059	0.373
7043	TGFB3	0.83	0.09	1155	0.372
55351	HSA250839	0.71	2.48e-03	1109	0.368
10455	PEC1	0.88	0.05	1059	0.366
58475	CFFM4	0.67	0.01	510	0.364
163	AP2B1	0.84	0.10	1155	0.363
79132	LGP2	0.67	1.70e-03	986	0.363

Poor prognosis					

55321	C20orf46	1.09	0.41	1137	-0.356
11082	ESM1	1.41	1.71e-03	1139	-0.357
9134	CCNE2	1.74	2.74e-06	1032	-0.357
54583	EGLN1	1.44	2.13e-03	981	-0.357
1058	CENPA	1.94	1.26e-09	1183	-0.358
9055	PRC1	1.87	1.03e-08	1137	-0.358
445815	AKAP2	1.01	0.95	928	-0.360
10874	NMU	1.51	1.12e-04	1183	-0.360
3488	IGFBP5	1.18	0.12	1155	-0.360
10531	MP1	1.08	0.52	893	-0.361
57110	LOC57110	1.50	2.16e-04	1109	-0.361
3488	IGFBP5	1.19	0.12	1155	-0.361
8577	TMEFF1	1.30	0.02	1077	-0.362
4175	MCM6	1.84	1.56e-08	1183	-0.364
643008	LOC643008	NA	NA	NA	-0.365
83879	CDCA7	1.02	0.93	387	-0.365
5984	RFC4	1.62	6.38e-06	1183	-0.366
23594	ORC6L	1.80	7.32e-08	1137	-0.366
6515	SLC2A3	1.12	0.29	1155	-0.366
57211	DKFZP564D0462	0.96	0.72	1004	-0.367
79791	FBXO31	0.85	0.13	1137	-0.367
1633	DCK	1.36	4.67e-03	1155	-0.368
51514	L2DTL	1.62	1.19e-05	1109	-0.369
1284	COL4A2	1.22	0.10	1004	-0.371
9833	KIAA0175	1.82	2.21e-08	1183	-0.371
92140	MTDH	1.32	0.01	1155	-0.373
51377	UCH37	1.19	0.11	1137	-0.374
51560	RAB6B	0.98	0.84	1109	-0.376
160897	GPR180	1.24	0.31	337	-0.379
79888	FLJ12443	1.31	0.02	1004	-0.381
8293	SERF1A	1.54	0.44	28	-0.383
8476	PK428	1.19	0.10	1183	-0.384
10403	HEC	1.34	7.04e-03	1183	-0.386
8833	GMPS	1.37	3.12e-03	1183	-0.386
1894	ECT2	1.59	1.70e-05	1137	-0.390
4318	MMP9	1.25	0.04	1183	-0.392
5019	OXCT	1.00	0.99	1183	-0.392
2781	GNAZ	1.08	0.49	1155	-0.396
2321	FLT1	1.05	0.71	857	-0.398
2131	EXT1	1.25	0.04	1183	-0.400
56942	DC13	1.80	4.69e-08	1137	-0.400
81624	DIAPH3	1.08	0.52	998	-0.405
81624	DIAPH3	1.08	0.52	998	-0.409
169714	QSOX2	1.57	0.04	343	-0.415
286052	LOC286052	NA	NA	NA	-0.424
51203	LOC51203	1.83	2.44e-08	1137	-0.425
81624	DIAPH3	1.08	0.52	998	-0.433
85453	TSPYL5	0.96	0.72	999	-0.527

DFS, disease-free survival; LNN, lymph node-negative.

### miRNAs associated with prognosis in breast cancer

**Figure 2:**
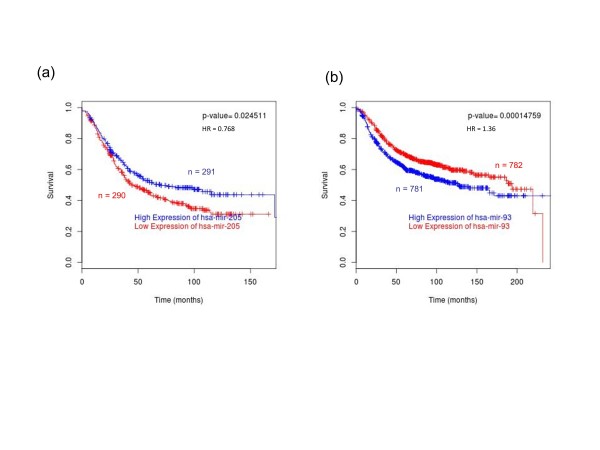
**miR-205 and miR-93 are associated with prognosis in breast cancer**. These figures were generated using *BreastMark *at http://glados.ucd.ie/BreastMark/index.html. (**a**) High expression of miR-205 is associated with good prognosis in breast cancer (HR = 0.768, *P*-value = 0.02, *n *= 581). (**b**) Low miR-93 expression is a marker of poor prognosis in breast cancer (HR = 1.36, P-value = 1.48e-4, *n *= 1563).

Decreased expression of miR-205 has previously been associated with poor prognosis in breast cancer, and miR-93 is highly expressed in high-grade tumours, that is, in tumours of patients who do poorly [10,34]; however, these studies were relatively small in scope (20 and 93 patients, respectively) [10,34]. To confirm these observations in a larger dataset and to test our approach, we examined the association of the host genes of these miRNAs with prognosis. The results for miR-205 and miR-93 can be seen in Figures 2(a) and 2(b), respectively. Following *BreastMark *analysis, high expression of the host gene of miR-205 is indeed associated with good prognosis (HR = 0.768, *P*-value = 0.02, *n *= 581) and high expression of host gene of miR-93 is associated with poor prognosis (HR = 1.34, *P*-value = 1.48e-04, *n *= 1,563). This confirms that miR-205 and miR-93 are robust markers of good and poor prognosis, respectively.

### Receptor tyrosine kinases associated with poor survival in the basal molecular subtype

RTKs are a large family of proteins involved in cell signalling with particular roles in growth, differentiation, adhesion, motility and death of cells [35]. A total of 58 kinases have been classified as receptor type and are listed in Additional file 2. Each of these kinases was assessed in the basal molecular subtype based on the three classifiers (ssp2003, ssp2006 and PAM50). Six of the kinases were significantly associated with poor prognosis in the basal subtype (EPHA5, FGFR1, FGFR3, VEGFR1, PDGFRβ and TIE1). The results are summarised on Table 5. As expected, the RTKs as a group have the potential to act as prognostic markers in this difficult-to-treat subtype of breast cancer. In particular, PDGFRβ would appear to be a strong marker of poor prognosis as it is significant across all three of the survival endpoints. This is not entirely unexpected as elevated levels of PDGFRβ have previously been associated with enhanced cell migration and invasion in breast cancer [36].

**Table 5:** Receptor tyrosine kinases associated with poor survival in the basal molecular subtype

Gene name	Gene description	Survival end point	Molecular classifier	Expression cut-off	Hazard ratio	*P*-value	Number
EPHA5	EPH receptor A5	OS	SSP2003	median	2.03	3.36e-03	233
		DFS	SSP2006	median	1.37	0.05	422
		OS	SSP2006	median	1.59	0.05	271
FGFR1	fibroblast growth factor receptor 1	DFS	SSP2006	High	1.43	0.02	465
		DFS	PAM50	High	1.36	0.05	408
FGFR3	fibroblast growth factor receptor 3	OS	SSP2003	High	1.63	0.04	273
		OS	SSP2003	Median	1.53	0.04	273
		OS	SSP2006	Median	1.62	0.01	323
		OS	PAM50	Median	1.54	0.03	293
VEGFR1	vascular endothelial growth factor receptor 1	DDFS	SSP2003	Low	1.84	0.05	320
		OS	SSP2003	Median	1.53	0.05	249
		OS	SSP2006	High	1.76	7.40e-03	284
		OS	SSP2006	Median	1.69	9.50e-03	284
		DDFS	SSP2006	Low	1.85	0.03	378
		DDFS	PAM50	Low	2.07	0.02	365
		OS	PAM50	High	1.61	0.04	261
		OS	PAM50	Median	1.61	0.03	261
PDGFRβ	platelet-derived growth factor receptor, beta polypeptide	DDFS	SSP2003	Median	1.88	1.64e-03	341
		DDFS	SSP2003	High	2.26	9.34e-04	341
		OS	SSP2003	Median	1.55	0.05	273
		DFS	SSP2006	Median	1.37	0.02	474
		OS	SSP2006	Median	1.72	5.84e-03	323
		OS	SSP2006	High	2.12	1.26e-03	323
		DDFS	SSP2006	High	1.76	0.01	423
		DFS	SSP2006	High	1.50	0.01	474
		DDFS	PAM50	Median	1.81	8.58e-04	393
		DDFS	PAM50	High	1.86	6.33e-03	393
		OS	PAM50	High	1.94	7.27e-03	293
		DFS	PAM50	High	1.58	7.56e-03	419
		DFS	PAM50	Median	1.38	0.02	419
		DDFS	PAM50	Low	1.45	0.04	393
TIE1	tyrosine kinase with immunoglobulin-like and EGF-like domains 1	OS	SSP2003	Median	1.63	0.02	273
		OS	SSP2006	Median	1.70	4.82e-03	323
		OS	PAM50	Median	1.56	0.03	293

DFS, disease-free survival; DDFS, distant disease-free survival; OS, overall survival.

## Discussion

*BreastMark *provides a user-friendly tool for examining putative prognostic markers in breast cancer. The value of the approach used here is based on its simplicity of operation and the statistical power gained through the combination of a large cohort of patients when compared to single microarray experiments. While it is not the first application which combines multiple public breast cancer datasets and performs a cross-dataset survival analysis [37-39], it is the first application which allows users to combine multiple prognostic markers across multiple microarray platforms without requiring complex adjustments for batch effects across different experiments/platforms. We are, therefore, not reliant on the suitability of the data transformation method chosen. Also, as the database is gene-centred, rather than probe-centred, we are not limited to the gene coverage of a particular platform. However, we are unable to examine the effects that splice variants may have on survival. While the analysis of splice variants is possible with some of the platforms used in this analysis, it is limited as most of these platforms predate the publication of the complete human genome. In summary, *BreastMark *allows the analysis of approximately 20,000 unique Entrez gene IDs in up to 4,739 samples. While some compromises were made in making the data gene centred, which negated the continuous nature of the gene expression information, our comparison with MammaPrint and onco*type *DX shows our approach to be robust.

In the case of onco*type *DX, our results suggest that some of the 16 oncogenes in the signature may not be necessary. It would appear that the five proliferation markers are sufficient for determining patient outcome, as these are the only genes with high HRs and are highly significant. This is consistent with previous findings [40-43]. In fact, combining the proliferation markers within *BreastMark *allows us to identify patients who will do even more poorly. However, when we combine the proliferation markers with most of the other 11 non-proliferation genes, the HR decreases and the Kaplan-Meier plots become less significant. This suggests that not only are all of the genes from this prognostic signature not required, but that our algorithm provides a useful reductionist approach to these complex prognostic signatures. This facilitates the elimination of superfluous markers and highlights those genes that are of the greatest relevance. Although MammaPrint uses a different approach to identify patients who will have a poor outcome, the use of our approach could substantially reduce the number of genes required in this prognostic signature, thus reducing the cost and the complexity of this signature.

After confirming the robustness of our algorithm we used it to examine the potential for inferring the prognostic ability of miRNAs from the gene expression data and to look at RTKs in the basal sub-type of breast cancer. The attraction of miRNA biology to cancer researchers arises from the potential of miRNAs to alter an entire pathway or, indeed, pathways. miRNAs have been heavily studied in breast cancer; however, their role as prognostic markers is not well characterised. There are only a few large-scale studies which incorporate miRNA profiling and detailed clinical data [10,44]. Despite the huge efforts required to compile these studies, their sample numbers are only in the hundreds and, therefore, not only do they have limited statistical power, they are also restricted in their ability to assess the rarer breast cancer subtypes. However, there is a wealth of gene expression data available with detailed clinical information which can be exploited by inferring miRNA activity from host gene expression.

Again, our approach gene centres the data and allows us to examine miRNAs as prognostic markers in breast cancer as a whole and within the molecular subtypes. We were able to confirm the results of smaller studies [10,45], which demonstrated that reduced expression of miR-205 (*n *= 20) and increased expression of miR-93 (*n *= 93) are associated with poor prognosis in breast cancer. As both of these studies were relatively small, their findings in isolation would be considered preliminary evidence. It should be noted, however, that not all miRNAs and host genes are co-expressed [14] and care needs to be taken when interpreting the results from *BreastMark*. This issue cannot be resolved until such time as there is a clearer picture of which miRNAs are co-expressed with their host genes (current estimates put it at approximately 70% [16]) and if those that are not significantly co-expressed do so in a disease/tissue specific manner or whether the miRNAs themselves are subject to some level of post-transcriptional regulation.

Tyrosine kinases are a large family of proteins involved in cell signalling with respect to growth, differentiation, adhesion, motility and death [35]. Of the 90 tyrosine kinases identified, 58 have been classified as receptor type. These 58 receptors can be further sub-divided into 20 families [46]. A number of families of RTKs have been implicated in the development of many cancers, including HER and IGFR families and so on through over-expression, amplification and/or aberrant signalling of the RTKs [47]. Using *BreastMark*, we were able to identify six RTKs that can be associated with poor prognosis in the basal subtype of breast cancer. These RTKs are putative markers of poor prognosis and are potential drug targets in this difficult-to-treat subtype of breast cancer. For example, increased expression of PDGFRβ has been associated with enhanced cell migration and invasion in breast cancer [31]; *BreastMark *identifies PDGFRβ as a marker of poor prognosis and this RTK has been shown to be inhibited by imatinib in phase I clinical trials [48]. In addition, imatinib has been investigated in advanced breast cancers expressing PDGFRβ [49]. Also, *BreastMark *identifies FGFR1 as a marker in the basal subtype of breast cancer, which has been previously shown as a marker of poor prognosis in the luminal subtypes [50].

## Conclusions

In this study, we have developed a simple user-friendly tool for examining putative gene/miRNA prognostic markers in breast cancer. The value of this tool is both in the simplicity of its design and the robustness of its approach. It is designed with non-bioinformatic research groups in mind and will be of great value in the preliminary assessment of putative biomarkers in breast cancer as a whole and within its molecular subtypes.

## Abbreviations

DDFS: distant disease-free survival; DFS: disease-free survival; ER: estrogen receptor; HER2: human epidermal growth factor receptor 2; HR: hazard ratio; LNN: lymph node-negative; miRNA: microRNA; OS: overall survival; PR: progesterone receptor; RS: recurrence score; RTK: receptor tyrosine kinase.

## Competing interests

The authors declare that they have no competing interests.

## Authors' contributions

SFM was involved in study conception, all experiments/data analyses and drafting of the manuscript. CC developed the website and had a significant role in data analysis and interpretation. PG and NOD performed the RTK analysis. WMG, MC, JC and STA were primary contributors to study conception, design and implementation. All authors read and approved the final manuscript.

## Supplementary Material

Additional file 1 **Table S1**. *BreastMark *results for Onco*type *DX 21-gene signature for LNN, ER-positive patients using DDFS as the survival end point. **Table S2**. *BreastMark *results for Onco*type *DX 21-gene signature for LNN, ER-positive patients using OS as the survival end point. **Table S3**. *BreastMark *results for the MammaPrint gene signature for LNN patients using DDFS as the survival end point. **Table S4**. *BreastMark *results for the MammaPrint gene signature for LNN patients using OS as the survival end point. Click here for file

Additional file 2 **The 58 RTKs examined using *BreastMark***. Click here for file

## References

[B1] Reis-Filho JS Pusztai L Gene expression profiling in breast cancer: classification, prognostication, and prediction Lancet 2011 15 1812 1823 10.1016/S0140-6736(11)61539-0 22098854

[B2] Ali S Coombes RC Endocrine-responsive breast cancer and strategies for combating resistance Nat Rev Cancer 2002 15 101 112 10.1038/nrc721 12635173

[B3] Sawyers C Targeted cancer therapy Nature 2004 15 294 297 10.1038/nature03095 15549090

[B4] Foulkes WD Smith IE Reis-Filho JS Triple-negative breast cancer N Engl J Med 2010 15 1938 1948 10.1056/NEJMra1001389 21067385

[B5] Sørlie T Tibshirani R Parker J Hastie T Marron JS Nobel A Deng S Johnsen H Pesich R Geisler S Demeter J Perou CM Lønning PE Brown PO Børresen-Dale A-L Botstein D Repeated observation of breast tumor subtypes in independent gene expression data sets Proceedings of the National Academy of Sciences of the United States of America 2003 15 8418 8423 10.1073/pnas.0932692100 12829800 PMC166244

[B6] Perou CM Sørlie T Eisen MB van de Rijn M Jeffrey SS Rees CA Pollack JR Ross DT Johnsen H Akslen LA Fluge O Pergamenschikov A Williams C Zhu SX Lønning PE Børresen-Dale AL Brown PO Botstein D Molecular portraits of human breast tumours Nature 2000 15 747 752 10.1038/35021093 10963602

[B7] Curtis C Shah SP Chin S-F Turashvili G Rueda OM Dunning MJ Speed D Lynch AG Samarajiwa S Yuan Y Gräf S Ha G Haffari G Bashashati A Russell R McKinney S Group M Langerød A Green A Provenzano E Wishart G Pinder S Watson P Markowetz F Murphy L Ellis I Purushotham A Børresen-Dale A-L Brenton JD Tavaré S Caldas C Aparicio S The genomic and transcriptomic architecture of 2,000 breast tumours reveals novel subgroups Nature 2012 15 346 352 22522925 10.1038/nature10983 PMC3440846

[B8] Bartel DP MicroRNAs: Target Recognition and Regulatory Functions Cell 2009 15 215 233 10.1016/j.cell.2009.01.002 19167326 PMC3794896

[B9] Krek A Grün D Poy MN Wolf R Rosenberg L Epstein EJ MacMenamin P da Piedade I Gunsalus KC Stoffel M Rajewsky N Combinatorial microRNA target predictions Nat Genet 2005 15 495 500 10.1038/ng1536 15806104

[B10] Blenkiron C Goldstein LD Thorne NP Spiteri I Chin S-F Dunning MJ Barbosa-Morais NL Teschendorff AE Green AR Ellis IO Tavaré S Caldas C Miska EA MicroRNA expression profiling of human breast cancer identifies new markers of tumor subtype Genome Biol 2007 15 R214 10.1186/gb-2007-8-10-r214 17922911 PMC2246288

[B11] Iorio MV Ferracin M Liu C-G Veronese A Spizzo R Sabbioni S Magri E Pedriali M Fabbri M Campiglio M Ménard S Palazzo JP Rosenberg A Musiani P Volinia S Nenci I Calin GA Querzoli P Negrini M Croce CM MicroRNA Gene Expression Deregulation in Human Breast Cancer Cancer Res 2005 15 7065 7070 10.1158/0008-5472.CAN-05-1783 16103053

[B12] Iorio MV Casalini P Tagliabue E Ménard S Croce CM MicroRNA profiling as a tool to understand prognosis, therapy response and resistance in breast cancer European Journal of Cancer 2008 15 2753 2759 10.1016/j.ejca.2008.09.037 19022662

[B13] Mattie MD Benz CC Bowers J Sensinger K Wong L Scott GK Fedele V Ginzinger D Getts R Haqq C Optimized high-throughput microRNA expression profiling provides novel biomarker assessment of clinical prostate and breast cancer biopsies Molecular Cancer 2006 15 24 10.1186/1476-4598-5-24 16784538 PMC1563474

[B14] Baskerville S Bartel DP Microarray profiling of microRNAs reveals frequent coexpression with neighboring miRNAs and host genes RNA 2005 15 241 247 10.1261/rna.7240905 15701730 PMC1370713

[B15] Rodriguez A Griffiths-Jones S Ashurst JL Bradley A Identification of Mammalian microRNA Host Genes and Transcription Units Genome Res 2004 15 1902 1910 10.1101/gr.2722704 15364901 PMC524413

[B16] Liang Y Ridzon D Wong L Chen C Characterization of microRNA expression profiles in normal human tissues BMC Genomics 0000 15 166 166 17565689 10.1186/1471-2164-8-166 PMC1904203

[B17] BreastMark: Breast Cancer Survival Analysis Tool http://glados.ucd.ie/BreastMark/index.html

[B18] Gene Expression Omnibus (GEO) Main page http://www.ncbi.nlm.nih.gov/geo/

[B19] Irizarry RA Hobbs B Collin F Beazer-Barclay YD Antonellis KJ Scherf U Speed TP Exploration, normalization, and summaries of high density oligonucleotide array probe level data Biostatistics 2003 15 249 264 10.1093/biostatistics/4.2.249 12925520

[B20] Bioconductor - Home http://www.bioconductor.org/

[B21] Yang YH Dudoit S Luu P Lin DM Peng V Ngai J Speed TP Normalization for cDNA microarray data: a robust composite method addressing single and multiple slide systematic variation Nucleic Acids Res 2002 15 e15 e15 10.1093/nar/30.4.e15 11842121 PMC100354

[B22] Maglott D Ostell J Pruitt KD Tatusova T Entrez Gene: gene-centered information at NCBI Nucleic Acids Res 2005 15 D54 58 10.1093/nar/gni052 15608257 PMC539985

[B23] Guberman JM Ai J Arnaiz O Baran J Blake A Baldock R Chelala C Croft D Cros A Cutts RJ Di Genova A Forbes S Fujisawa T Gadaleta E Goodstein DM Gundem G Haggarty B Haider S Hall M Harris T Haw R Hu S Hubbard S Hsu J Iyer V Jones P Katayama T Kinsella R Kong L Lawson D Liang Y Lopez-Bigas N Luo J Lush M Mason J Moreews F Ndegwa N Oakley D Perez-Llamas C Primig M Rivkin E Rosanoff S Shepherd R Simon R Skarnes B Smedley D Sperling L Spooner W Stevenson P Stone K Teague J Wang J Wang J Whitty B Wong DT Wong-Erasmus M Yao L Youens-Clark K Yung C Zhang J Kasprzyk A BioMart Central Portal: an open database network for the biological community Database 2011 15 bar041 bar041 10.1093/database/bar041 21930507 PMC3263598

[B24] BioMart http://www.biomart.org/

[B25] Rainer J Ploner C Jesacher S Ploner A Eduardoff M Mansha M Wasim M Panzer-Grumayer R Trajanoski Z Niederegger H Kofler R Glucocorticoid-regulated microRNAs and mirtrons in acute lymphoblastic leukemia Leukemia 2009 15 746 752 10.1038/leu.2008.370 19148136

[B26] Bioconductor - genefu http://www.bioconductor.org/packages/release/bioc/html/genefu.html

[B27] Hu Z Fan C Oh D Marron J He X Qaqish B Livasy C Carey L Reynolds E Dressler L Nobel A Parker J Ewend M Sawyer L Wu J Liu Y Nanda R Tretiakova M Orrico A Dreher D Palazzo J Perreard L Nelson E Mone M Hansen H Mullins M Quackenbush J Ellis M Olopade O Bernard P Perou C The molecular portraits of breast tumors are conserved across microarray platforms BMC Genomics 2006 15 96 10.1186/1471-2164-7-96 16643655 PMC1468408

[B28] Parker JS Mullins M Cheang MCU Leung S Voduc D Vickery T Davies S Fauron C He X Hu Z Quackenbush JF Stijleman IJ Palazzo J Marron JS Nobel AB Mardis E Nielsen TO Ellis MJ Perou CM Bernard PS Supervised Risk Predictor of Breast Cancer Based on Intrinsic Subtypes Journal of Clinical Oncology 2009 15 1160 1167 10.1200/JCO.2008.18.1370 19204204 PMC2667820

[B29] The Comprehensive R Archive Network http://cran.r-project.org/

[B30] Paik S Shak S Tang G Kim C Baker J Cronin M Baehner FL Walker MG Watson D Park T Hiller W Fisher ER Wickerham DL Bryant J Wolmark N A multigene assay to predict recurrence of tamoxifen-treated, node-negative breast cancer N Engl J Med 2004 15 2817 2826 10.1056/NEJMoa041588 15591335

[B31] Chang HY Nuyten DSA Sneddon JB Hastie T Tibshirani R Sørlie T Dai H He YD van't Veer LJ Bartelink H van de Rijn M Brown PO van de Vijver MJ Robustness, scalability, and integration of a wound-response gene expression signature in predicting breast cancer survival Proc Natl Acad Sci USA 2005 15 3738 3743 10.1073/pnas.0409462102 15701700 PMC548329

[B32] Paik S Shak S Tang G Kim C Baker J Cronin M Baehner FL Walker MG Watson D Park T Hiller W Fisher ER Wickerham DL Bryant J Wolmark N A multigene assay to predict recurrence of tamoxifen-treated, node-negative breast cancer N Engl J Med 2004 15 2817 2826 10.1056/NEJMoa041588 15591335

[B33] Van 't Veer LJ Dai H van de Vijver MJ He YD Hart AAM Mao M Peterse HL van der Kooy K Marton MJ Witteveen AT Schreiber GJ Kerkhoven RM Roberts C Linsley PS Bernards R Friend SH Gene expression profiling predicts clinical outcome of breast cancer Nature 2002 15 530 536 10.1038/415530a 11823860

[B34] Sempere LF Christensen M Silahtaroglu A Bak M Heath CV Schwartz G Wells W Kauppinen S Cole CN Altered MicroRNA Expression Confined to Specific Epithelial Cell Subpopulations in Breast Cancer Cancer Research 2007 15 11612 11620 10.1158/0008-5472.CAN-07-5019 18089790

[B35] Choura M Rebaï A Receptor tyrosine kinases: from biology to pathology J Recept Signal Transduct Res 2011 15 387 394 10.3109/10799893.2011.625425 22040163

[B36] Campbell CI Moorehead RA Mammary tumors that become independent of the type I insulin-like growth factor receptor express elevated levels of platelet-derived growth factor receptors BMC Cancer 2011 15 480 10.1186/1471-2407-11-480 22070644 PMC3254084

[B37] Györffy B Lanczky A Eklund AC Denkert C Budczies J Li Q Szallasi Z An online survival analysis tool to rapidly assess the effect of 22,277 genes on breast cancer prognosis using microarray data of 1,809 patients Breast Cancer Res Treat 2009 15 725 731 20020197 10.1007/s10549-009-0674-9

[B38] Jézéquel P Campone M Gouraud W Guérin-Charbonnel C Leux C Ricolleau G Campion L bc-GenExMiner: an easy-to-use online platform for gene prognostic analyses in breast cancer Breast Cancer Res Treat 2012 15 765 775 10.1007/s10549-011-1457-7 21452023

[B39] Clarke C Madden SF Doolan P Aherne ST Joyce H O'Driscoll L Gallagher WM Hennessy BT Moriarty M Crown J Kennedy S Clynes M Correlating transcriptional networks to breast cancer survival: a large-scale coexpression analysis Carcinogenesis 2013 15 2300 2308 10.1093/carcin/bgt208 23740839

[B40] Lee JJ Shen J Is the Oncotype DX assay necessary in strongly estrogen receptor-positive breast cancers? Am Surg 2011 15 1364 1367 22127090

[B41] Allison KH Kandalaft PL Sitlani CM Dintzis SM Gown AM Routine pathologic parameters can predict Oncotype DX recurrence scores in subsets of ER positive patients: who does not always need testing? Breast Cancer Res Treat 2012 15 413 424 10.1007/s10549-011-1416-3 21369717

[B42] Paik S Is gene array testing to be considered routine now? Breast 2011 15 Suppl 3 S87 91 22015300 10.1016/S0960-9776(11)70301-0

[B43] Sahebjam S Aloyz R Pilavdzic D Brisson M-L Ferrario C Bouganim N Cohen V Miller WH Panasci LC Ki 67 is a major, but not the sole determinant of Oncotype Dx recurrence score Br J Cancer 2011 15 1342 1345 10.1038/bjc.2011.402 21970880 PMC3241562

[B44] Buffa FM Camps C Winchester L Snell CE Gee HE Sheldon H Taylor M Harris AL Ragoussis J microRNA associated progression pathways and potential therapeutic targets identified by integrated mRNA and microRNA expression profiling in breast cancer Cancer Research 2011 10.1158/0008-5472.CAN-11-0489 21737487

[B45] Sempere LF Freemantle S Pitha-Rowe I Moss E Dmitrovsky E Ambros V Expression profiling of mammalian microRNAs uncovers a subset of brain-expressed microRNAs with possible roles in murine and human neuronal differentiation Genome Biol 2004 15 R13 10.1186/gb-2004-5-3-r13 15003116 PMC395763

[B46] Robinson DR Wu YM Lin SF The protein tyrosine kinase family of the human genome Oncogene 2000 15 5548 5557 10.1038/sj.onc.1203957 11114734

[B47] Zwick E Bange J Ullrich A Receptor tyrosine kinase signalling as a target for cancer intervention strategies Endocr Relat Cancer 2001 15 161 173 10.1677/erc.0.0080161 11566607

[B48] Al-Batran S-E Atmaca A Schleyer E Pauligk C Hosius C Ehninger G Jäger E Imatinib mesylate for targeting the platelet-derived growth factor beta receptor in combination with fluorouracil and leucovorin in patients with refractory pancreatic, bile duct, colorectal, or gastric cancer--a dose-escalation Phase I trial Cancer 2007 15 1897 1904 10.1002/cncr.22622 17377918

[B49] Cristofanilli M Morandi P Krishnamurthy S Reuben JM Lee B-N Francis D Booser DJ Green MC Arun BK Pusztai L Lopez A Islam R Valero V Hortobagyi GN Imatinib mesylate (Gleevec^®^) in advanced breast cancer-expressing C-Kit or PDGFR-β: clinical activity and biological correlations Ann Oncol 2008 15 1713 1719 10.1093/annonc/mdn352 18515258 PMC2735063

[B50] Turner N Pearson A Sharpe R Lambros M Geyer F Lopez-Garcia MA Natrajan R Marchio C Iorns E Mackay A Gillett C Grigoriadis A Tutt A Reis-Filho JS Ashworth A FGFR1 Amplification Drives Endocrine Therapy Resistance and Is a Therapeutic Target in Breast Cancer Cancer Res 2010 15 2085 2094 10.1158/0008-5472.CAN-09-3746 20179196 PMC2832818

[B51] Anders CK Acharya CR Hsu DS Broadwater G Garman K Foekens JA Zhang Y Wang Y Marcom K Marks JR Mukherjee S Nevins JR Blackwell KL Potti A Age-Specific Differences in Oncogenic Pathway Deregulation Seen in Human Breast Tumors PLoS ONE 2008 15 e1373 10.1371/journal.pone.0001373 18167534 PMC2148101

[B52] Bild AH Yao G Chang JT Wang Q Potti A Chasse D Joshi M-B Harpole D Lancaster JM Berchuck A Olson JA Marks JR Dressman HK West M Nevins JR Oncogenic pathway signatures in human cancers as a guide to targeted therapies Nature 2006 15 353 357 10.1038/nature04296 16273092

[B53] Bos PD Zhang XH-F Nadal C Shu W Gomis RR Nguyen DX Minn AJ van de Vijver MJ Gerald WL Foekens JA Massagué J Genes that mediate breast cancer metastasis to the brain Nature 2009 15 1005 1009 10.1038/nature08021 19421193 PMC2698953

[B54] Calabrò A Beissbarth T Kuner R Stojanov M Benner A Asslaber M Ploner F Zatloukal K Samonigg H Poustka A Sültmann H Effects of infiltrating lymphocytes and estrogen receptor on gene expression and prognosis in breast cancer Breast Cancer Res Treat 2008 15 69 77 18592372 10.1007/s10549-008-0105-3

[B55] Chin K DeVries S Fridlyand J Spellman PT Roydasgupta R Kuo W-L Lapuk A Neve RM Qian Z Ryder T Chen F Feiler H Tokuyasu T Kingsley C Dairkee S Meng Z Chew K Pinkel D Jain A Ljung BM Esserman L Albertson DG Waldman FM Gray JW Genomic and transcriptional aberrations linked to breast cancer pathophysiologies Cancer Cell 2006 15 529 541 10.1016/j.ccr.2006.10.009 17157792

[B56] Chanrion M Negre V Fontaine H Salvetat N Bibeau F Grogan GM Mauriac L Katsaros D Molina F Theillet C Darbon J-M A gene expression signature that can predict the recurrence of tamoxifen-treated primary breast cancer Clin Cancer Res 2008 15 1744 1752 10.1158/1078-0432.CCR-07-1833 18347175 PMC2912334

[B57] Desmedt C Piette F Loi S Wang Y Lallemand F Haibe-Kains B Viale G Delorenzi M Zhang Y d' Assignies MS Bergh J Lidereau R Ellis P Harris AL Klijn JGM Foekens JA Cardoso F Piccart MJ Buyse M Sotiriou C Strong Time Dependence of the 76-Gene Prognostic Signature for Node-Negative Breast Cancer Patients in the TRANSBIG Multicenter Independent Validation Series Clinical Cancer Research 2007 15 3207 3214 10.1158/1078-0432.CCR-06-2765 17545524

[B58] Desmedt C Giobbie-Hurder A Neven P Paridaens R Christiaens M-R Smeets A Lallemand F Haibe-Kains B Viale G Gelber R Piccart M Sotiriou C The Gene expression Grade Index: a potential predictor of relapse for endocrine-treated breast cancer patients in the BIG 1-98 trial BMC Medical Genomics 2009 15 40 10.1186/1755-8794-2-40 19573224 PMC2713270

[B59] Hatzis C Pusztai L Valero V Booser DJ Esserman L Lluch A Vidaurre T Holmes F Souchon E Wang H Martin M Cotrina J Gomez H Hubbard R Chacón JI Ferrer-Lozano J Dyer R Buxton M Gong Y Wu Y Ibrahim N Andreopoulou E Ueno NT Hunt K Yang W Nazario A DeMichele A O'Shaughnessy J Hortobagyi GN Symmans WF A genomic predictor of response and survival following taxane-anthracycline chemotherapy for invasive breast cancer JAMA 2011 15 1873 1881 10.1001/jama.2011.593 21558518 PMC5638042

[B60] Heikkinen T Greco D Pelttari LM Tommiska J Vahteristo P Heikkilä P Blomqvist C Aittomäki K Nevanlinna H Variants on the promoter region of PTEN affect breast cancer progression and patient survival Breast Cancer Res 2011 15 R130 10.1186/bcr3076 22171747 PMC3326572

[B61] Kao K-J Chang K-M Hsu H-C Huang AT Correlation of microarray-based breast cancer molecular subtypes and clinical outcomes: implications for treatment optimization BMC Cancer 2011 15 143 10.1186/1471-2407-11-143 21501481 PMC3094326

[B62] Kok M Linn SC Van Laar RK Jansen MPHM van den Berg TM Delahaye LJMJ Glas AM Peterse JL Hauptmann M Foekens JA Klijn JGM Wessels LFA Van't Veer LJ Berns EMJJ Comparison of gene expression profiles predicting progression in breast cancer patients treated with tamoxifen Breast Cancer Res Treat 2009 15 275 283 10.1007/s10549-008-9939-y 18311582

[B63] Loi S Haibe-Kains B Desmedt C Wirapati P Lallemand F Tutt A Gillet C Ellis P Ryder K Reid J Daidone M Pierotti M Berns E Jansen M Foekens J Delorenzi M Bontempi G Piccart M Sotiriou C Predicting prognosis using molecular profiling in estrogen receptor-positive breast cancer treated with tamoxifen BMC Genomics 2008 15 239 10.1186/1471-2164-9-239 18498629 PMC2423197

[B64] Ma X-J Wang Z Ryan PD Isakoff SJ Barmettler A Fuller A Muir B Mohapatra G Salunga R Tuggle JT Tran Y Tran D Tassin A Amon P Wang W Wang W Enright E Stecker K Estepa-Sabal E Smith B Younger J Balis U Michaelson J Bhan A Habin K Baer TM Brugge J Haber DA Erlander MG Sgroi DC A two-gene expression ratio predicts clinical outcome in breast cancer patients treated with tamoxifen Cancer Cell 2004 15 607 616 10.1016/j.ccr.2004.05.015 15193263

[B65] Miller LD Smeds J George J Vega VB Vergara L Ploner A Pawitan Y Hall P Klaar S Liu ET Bergh J An expression signature for p53 status in human breast cancer predicts mutation status, transcriptional effects, and patient survival Proceedings of the National Academy of Sciences of the United States of America 2005 15 13550 13555 10.1073/pnas.0506230102 16141321 PMC1197273

[B66] Nagalla S Chou JW Willingham MC Ruiz J Vaughn JP Dubey P Lash TL Hamilton-Dutoit SJ Bergh J Sotiriou C Black MA Miller LD Interactions between immunity, proliferation and molecular subtype in breast cancer prognosis Genome Biology 2013 15 R34 10.1186/gb-2013-14-4-r34 23618380 PMC3798758

[B67] Pawitan Y Bjohle J Amler L Borg A-L Egyhazi S Hall P Han X Holmberg L Huang F Klaar S Liu E Miller L Nordgren H Ploner A Sandelin K Shaw P Smeds J Skoog L Wedren S Bergh J Gene expression profiling spares early breast cancer patients from adjuvant therapy: derived and validated in two population-based cohorts Breast Cancer Research 2005 15 R953 R964 10.1186/bcr1325 16280042 PMC1410752

[B68] Sabatier R Finetti P Cervera N Lambaudie E Esterni B Mamessier E Tallet A Chabannon C Extra J-M Jacquemier J Viens P Birnbaum D Bertucci F A gene expression signature identifies two prognostic subgroups of basal breast cancer Breast Cancer Res Treat 2010 15 407 420 20490655 10.1007/s10549-010-0897-9

[B69] Schmidt M Böhm D von Törne C Steiner E Puhl A Pilch H Lehr H Hengstler J Kölbl H Gehrmann M The humoral immune system has a key prognostic impact in node-negative breast cancer Cancer research 2008 15 5405 5413 10.1158/0008-5472.CAN-07-5206 18593943

[B70] Sircoulomb F Bekhouche I Finetti P Adélaïde J Ben Hamida A Bonansea J Raynaud S Innocenti C Charafe-Jauffret E Tarpin C Ben Ayed F Viens P Jacquemier J Bertucci F Birnbaum D Chaffanet M Genome profiling of ERBB2-amplified breast cancers BMC Cancer 2010 15 539 10.1186/1471-2407-10-539 20932292 PMC2958950

[B71] Wang Y Klijn JG Zhang Y Sieuwerts AM Look MP Yang F Talantov D Timmermans M Meijer-van Gelder ME Yu J Jatkoe T Berns EM Atkins D Foekens JA Gene-expression profiles to predict distant metastasis of lymph-node-negative primary breast cancer The Lancet 2005 15 671 679 10.1016/S0140-6736(05)17947-1 15721472

[B72] Zhang Y Sieuwerts AM McGreevy M Casey G Cufer T Paradiso A Harbeck N Span PN Hicks DG Crowe J Tubbs RR Budd GT Lyons J Sweep FCGJ Schmitt M Schittulli F Golouh R Talantov D Wang Y Foekens JA The 76-gene signature defines high-risk patients that benefit from adjuvant tamoxifen therapy Breast Cancer Res Treat 2009 15 303 309 10.1007/s10549-008-0183-2 18821012

