# Supplementary material for: BreastMark: An Integrated Approach to Mining Publicly Available Transcriptomic Datasets Relating to Breast Cancer Outcome
Source: Breast Cancer Res. 2013 Jul 2;15(4):R52. doi: 10.1186/bcr3444 (PMC3978487; doi:10.1186/bcr3444)
Supplement: Additional file 1 — Table S1. BreastMark results for Oncotype DX 21-gene signature for LNN, ER-positive patients using DDFS as the survival end point. Table S2. BreastMark results for Oncotype DX 21-gene signature for LNN, ER-positive patients using OS as the survival end point. Table S3. BreastMark results for the MammaPrint gene signature for LNN patients using DDFS as the survival end point. Table S4. BreastMark results for the MammaPrint gene signature for LNN patients using OS as the survival end point. [file bcr3444-S1.DOC]

**Table S1**. *BreastMark* results for Onco*type* DX 21-gene signature, for LNN, ER-positive patients using DDFS as the survival end point.

|  | **Gene Symbol** | ***BreastMark***  **Hazard Ratio** | ***BreastMark* HR**  **P-value** | **Sample Number** | **RS Weighting** |
| --- | --- | --- | --- | --- | --- |
| Proliferation | KI67 | 1.37 | 0.14 | 512 | +1.04 |
|  | STK15 | 1.59 | 0.03 | 512 |  |
|  | Survivin | 2.02 | 8.14e-04 | 512 |  |
|  | CCNB1 | 1.55 | 0.04 | 512 |  |
|  | MYBL2 | 1.40 | 0.11 | 512 |  |
| Invasion | MMP11 | 1.55 | 0.04 | 512 | +0.1 |
|  | CTSL2 | 1.74 | 8.56e-03 | 512 |  |
| HER2 | GRB7 | 1.41 | 0.11 | 512 | +0.47 |
|  | HER2 | 1.17 | 0.46 | 512 |  |
| ER | ER | 1.24 | 0.33 | 512 | -0.34 |
|  | PGR | 1.03 | 0.91 | 512 |  |
|  | BCL2 | 0.83 | 0.36 | 512 |  |
|  | SCUBE2 | 0.47 | 2.32e-03 | 406 |  |
| Other | GSTM1 | 0.92 | 0.71 | 368 | -0.08 |
|  | CD68 | 1.02 | 0.94 | 512 | +0.05 |
|  | BAG1 | 0.84 | 0.40 | 512 | -0.07 |

**Table S2.** *BreastMark* results for Onco*type* DX 21-gene signature, for LNN, ER-positive patients using OS as the survival end point.

|  | **Gene Symbol** | ***BreastMark***  **Hazard Ratio** | ***BreastMark* HR**  **P-value** | **Sample Number** | **RS Weighting** |
| --- | --- | --- | --- | --- | --- |
| Proliferation | KI67 | 1.44 | 0.15 | 411 | +1.04 |
|  | STK15 | 1.60 | 0.06 | 411 |  |
|  | Survivin | 2.09 | 3.02e-03 | 411 |  |
|  | CCNB1 | 2.04 | 0.02 | 302 |  |
|  | MYBL2 | 1.99 | 5.90e-03 | 411 |  |
| Invasion | MMP11 | 2.02 | 9.12e-03 | 411 | +0.1 |
|  | CTSL2 | 1.65 | 0.06 | 411 |  |
| HER2 | GRB7 | 1.96 | 7.45e-03 | 411 | +0.47 |
|  | HER2 | 1.39 | 0.20 | 411 |  |
| ER | ER | 0.89 | 0.64 | 411 | -0.34 |
|  | PGR | 0.64 | 0.08 | 411 |  |
|  | BCL2 | 0.79 | 0.35 | 411 |  |
|  | SCUBE2 | 0.55 | 0.12 | 195 |  |
| Other | GSTM1 | 0.68 | 0.15 | 336 | -0.08 |
|  | CD68 | 1.02 | 0.93 | 411 | +0.05 |
|  | BAG1 | 1.18 | 0.52 | 411 | -0.07 |

**Table S3.** *BreastMark* results for the MammaPrint gene signature for LNN patients using DDFS as the survival end point.

| **Entrez Gene ID** | **Gene Name** | **Hazard Ratio** | **P-value** | **Sample Number** | **Correlation with prognosis** |
| --- | --- | --- | --- | --- | --- |
| *Good Prognosis* | | | | | |
| 8659 | ALDH4 | 0.83 | 0.23 | 823 | 0.421 |
| 8817 | FGF18 | 1.06 | 0.70 | 900 | 0.411 |
| 27113 | BBC3 | 1.14 | 0.45 | 775 | 0.407 |
| 57593 | KIAA1442 | NA | NA | NA | 0.402 |
| 57758 | CEGP1 | 0.54 | 1.78e-04 | 772 | 0.400 |
| 146923 | RUNDC1 | 1.03 | 0.92 | 276 | 0.390 |
| 8840 | WISP1 | 0.72 | 0.03 | 900 | 0.384 |
| 2947 | GSTM3 | 0.90 | 0.45 | 900 | 0.380 |
| 151126 | ZNF533 | 1.32 | 0.30 | 276 | 0.375 |
| 146760 | RTN4RL1 | 0.86 | 0.60 | 199 | 0.374 |
| 10455 | PECI | 0.64 | 0.02 | 529 | 0.373 |
| 7043 | TGFB3 | 0.68 | 0.01 | 900 | 0.372 |
| 55351 | HSA250839 | 0.83 | 0.20 | 900 | 0.368 |
| 10455 | PEC1 | 0.72 | 0.03 | 823 | 0.366 |
| 58475 | CFFM4 | 0.94 | 0.83 | 276 | 0.364 |
| 163 | AP2B1 | 0.83 | 0.20 | 900 | 0.363 |
| 79132 | LGP2 | 1.01 | 0.94 | 900 | 0.363 |
| *Poor Prognosis* | | | | | |
| 55321 | C20orf46 | 0.92 | 0.57 | 900 | -0.356 |
| 11082 | ESM1 | 1.38 | 0.03 | 900 | -0.357 |
| 9134 | CCNE2 | 1.99 | 3.65e-05 | 775 | -0.357 |
| 54583 | EGLN1 | 1.46 | 0.01 | 900 | -0.357 |
| 1058 | CENPA | 2.21 | 4.15e-06 | 775 | -0.358 |
| 9055 | PRC1 | 1.53 | 4.41e-03 | 900 | -0.358 |
| 445815 | AKAP2 | 1.25 | 0.24 | 650 | -0.360 |
| 10874 | NMU | 1.71 | 2.69e-04 | 900 | -0.360 |
| 3488 | IGFBP5 | 1.16 | 0.32 | 900 | -0.360 |
| 10531 | MP1 | 1.05 | 0.76 | 823 | -0.361 |
| 57110 | LOC57110 | 1.32 | 0.06 | 900 | -0.361 |
| 3488 | IGFBP5 | 1.16 | 0.18 | 900 | -0.361 |
| 8577 | TMEFF1 | 1.43 | 0.02 | 823 | -0.362 |
| 4175 | MCM6 | 1.60 | 1.56e-03 | 900 | -0.364 |
| 643008 | LOC643008 | NA | NA | NA | -0.365 |
| 83879 | CDCA7 | 1.12 | 0.66 | 276 | -0.365 |
| 5984 | RFC4 | 1.70 | 4.06e-04 | 900 | -0.366 |
| 23594 | ORC6L | 1.57 | 2.59e-03 | 900 | -0.366 |
| 6515 | SLC2A3 | 0.86 | 0.30 | 900 | -0.366 |
| 57211 | DKFZP564D0462 | 1.09 | 0.56 | 900 | -0.367 |
| 79791 | FBXO31 | 0.90 | 0.47 | 900 | -0.367 |
| 1633 | DCK | 1.38 | 0.03 | 900 | -0.368 |
| 51514 | L2DTL | 1.55 | 3.33e-03 | 900 | -0.369 |
| 1284 | COL4A2 | 1.11 | 0.47 | 900 | -0.371 |
| 9833 | KIAA0175 | 1.74 | 1.95e-04 | 900 | -0.371 |
| 92140 | MTDH | 0.98 | 0.90 | 849 | -0.373 |
| 51377 | UCH37 | 1.42 | 0.02 | 900 | -0.374 |
| 51560 | RAB6B | 0.96 | 0.78 | 900 | -0.376 |
| 160897 | GPR180 | 2.03 | 0.01 | 276 | -0.379 |
| 79888 | FLJ12443 | 1.00 | 0.98 | 900 | -0.381 |
| 8293 | SERF1A | 1.54 | 0.44 | 28 | -0.383 |
| 8476 | PK428 | 1.14 | 0.36 | 900 | -0.384 |
| 10403 | HEC | 1.15 | 0.35 | 900 | -0.386 |
| 8833 | GMPS | 1.70 | 3.67e-04 | 900 | -0.386 |
| 1894 | ECT2 | 1.58 | 2.23e-03 | 900 | -0.390 |
| 4318 | MMP9 | 1.11 | 0.48 | 900 | -0.392 |
| 5019 | OXCT | 0.83 | 0.19 | 900 | -0.392 |
| 2781 | GNAZ | 0.92 | 0.59 | 900 | -0.396 |
| 2321 | FLT1 | 0.96 | 0.81 | 749 | -0.398 |
| 2131 | EXT1 | 0.93 | 0.65 | 900 | -0.400 |
| 56942 | DC13 | 1.97 | 6.58e-06 | 900 | -0.400 |
| 81624 | DIAPH3 | 0.78 | 0.19 | 823 | -0.405 |
| 81624 | DIAPH3 | 0.78 | 0.19 | 823 | -0.409 |
| 169714 | QSOX2 | 1.16 | 0.57 | 276 | -0.415 |
| 286052 | LOC286052 | NA | NA | NA | -0.424 |
| 51203 | LOC51203 | 1.35 | 0.05 | 900 | -0.425 |
| 81624 | DIAPH3 | 0.78 | 0.19 | 823 | -0.433 |
| 85453 | TSPYL5 | 0.86 | 0.33 | 900 | -0.527 |

**Table S4.** *BreastMark* results for the MammaPrint gene signature for LNN patients using OS as the survival end point.

| **Entrez Gene ID** | **Gene Name** | **Hazard Ratio** | **P-value** | **Sample Number** | **Correlation with prognosis** |
| --- | --- | --- | --- | --- | --- |
| *Good Prognosis* | | | | | |
| 8659 | ALDH4 | 0.71 | 0.09 | 467 | 0.421 |
| 8817 | FGF18 | 0.79 | 0.23 | 545 | 0.411 |
| 27113 | BBC3 | 1.03 | 0.92 | 394 | 0.407 |
| 57593 | KIAA1442 | NA | NA | 16 | 0.402 |
| 57758 | CEGP1 | 0.57 | 0.10 | 255 | 0.400 |
| 146923 | RUNDC1 | 0.35 | 0.01 | 155 | 0.390 |
| 8840 | WISP1 | 0.66 | 0.03 | 540 | 0.384 |
| 2947 | GSTM3 | 0.52 | 8.09e-04 | 545 | 0.380 |
| 151126 | ZNF533 | 1.24 | 0.58 | 150 | 0.375 |
| 146760 | RTN4RL1 | 1.54 | 0.30 | 77 | 0.374 |
| 10455 | PECI | 0.67 | 0.04 | 467 | 0.373 |
| 7043 | TGFB3 | 0.52 | 1.17e-03 | 545 | 0.372 |
| 55351 | HSA250839 | 0.50 | 5.85e-04 | 545 | 0.368 |
| 10455 | PEC1 | 0.63 | 0.03 | 377 | 0.366 |
| 58475 | CFFM4 | 0.64 | 0.06 | 306 | 0.364 |
| 163 | AP2B1 | 0.69 | 0.06 | 545 | 0.363 |
| 79132 | LGP2 | 0.77 | 0.37 | 394 | 0.363 |
| *Poor Prognosis* | | | | | |
| 55321 | C20orf46 | 1.62 | 0.01 | 545 | -0.356 |
| 11082 | ESM1 | 1.89 | 1.05e-03 | 529 | -0.357 |
| 9134 | CCNE2 | 1.4 | 0.18 | 394 | -0.357 |
| 54583 | EGLN1 | 1.54 | 0.09 | 389 | -0.357 |
| 1058 | CENPA | 1.90 | 1.24e-03 | 545 | -0.358 |
| 9055 | PRC1 | 2.19 | 7.79e-05 | 545 | -0.358 |
| 445815 | AKAP2 | 1.66 | 0.01 | 540 | -0.360 |
| 10874 | NMU | 2.11 | 9.93e-05 | 545 | -0.360 |
| 3488 | IGFBP5 | 1.17 | 0.42 | 545 | -0.360 |
| 10531 | MP1 | 0.44 | 0.02 | 255 | -0.361 |
| 57110 | LOC57110 | 1.54 | 0.03 | 545 | -0.361 |
| 3488 | IGFBP5 | 1.17 | 0.42 | 545 | -0.361 |
| 8577 | TMEFF1 | 1.52 | 0.04 | 467 | -0.362 |
| 4175 | MCM6 | 2.07 | 2.07e-04 | 545 | -0.364 |
| 643008 | LOC643008 | NA | NA | NA | -0.365 |
| 83879 | CDCA7 | 0.51 | 0.12 | 155 | -0.365 |
| 5984 | RFC4 | 1.79 | 2.98e-03 | 545 | -0.366 |
| 23594 | ORC6L | 2.32 | 1.65e-05 | 545 | -0.366 |
| 6515 | SLC2A3 | 1.33 | 0.15 | 545 | -0.366 |
| 57211 | DKFZP564D0462 | 0.55 | 0.03 | 394 | -0.367 |
| 79791 | FBXO31 | 1.17 | 0.43 | 545 | -0.367 |
| 1633 | DCK | 1.67 | 8.97e-03 | 545 | -0.368 |
| 51514 | L2DTL | 1.34 | 0.14 | 545 | -0.369 |
| 1284 | COL4A2 | 1.06 | 0.82 | 394 | -0.371 |
| 9833 | KIAA0175 | 2.15 | 8.62e-05 | 545 | -0.371 |
| 92140 | MTDH | 1.61 | 0.02 | 545 | -0.373 |
| 51377 | UCH37 | 1.15 | 0.49 | 545 | -0.374 |
| 51560 | RAB6B | 2.38 | 6.68e-06 | 545 | -0.376 |
| 160897 | GPR180 | 1.71 | 0.16 | 133 | -0.379 |
| 79888 | FLJ12443 | 1.94 | 8.08e-03 | 394 | -0.381 |
| 8293 | SERF1A | 1.54 | 0.44 | 28 | -0.383 |
| 8476 | PK428 | 1.12 | 0.56 | 545 | -0.384 |
| 10403 | HEC | 2.08 | 2.26e-04 | 545 | -0.386 |
| 8833 | GMPS | 2.07 | 2.01e-04 | 545 | -0.386 |
| 1894 | ECT2 | 1.48 | 0.04 | 545 | -0.390 |
| 4318 | MMP9 | 1.20 | 0.36 | 545 | -0.392 |
| 5019 | OXCT | 1.15 | 0.48 | 545 | -0.392 |
| 2781 | GNAZ | 1.71 | 6.02e-03 | 545 | -0.396 |
| 2321 | FLT1 | 1.19 | 0.38 | 451 | -0.398 |
| 2131 | EXT1 | 1.85 | 1.91e-03 | 545 | -0.400 |
| 56942 | DC13 | 2.48 | 4.84e-06 | 545 | -0.400 |
| 81624 | DIAPH3 | 3.24 | 1.23e-07 | 406 | -0.405 |
| 81624 | DIAPH3 | 3.24 | 1.23e-07 | 406 | -0.409 |
| 169714 | QSOX2 | 0.95 | 0.89 | 139 | -0.415 |
| 286052 | LOC286052 | NA | NA | NA | -0.424 |
| 51203 | LOC51203 | 2.08 | 1.92e-04 | 545 | -0.425 |
| 81624 | DIAPH3 | 3.24 | 1.23e-07 | 406 | -0.433 |
| 85453 | TSPYL5 | 0.85 | 0.52 | 389 | -0.527 |
